# Cell-Based Metabolomics Approach for Anticipating and Investigating Cytotoxicity of Gold Nanorods

**DOI:** 10.3390/foods11223569

**Published:** 2022-11-09

**Authors:** Jian Ji, Jiadi Sun, Yinzhi Zhang, Xiulan Sun

**Affiliations:** 1 State Key Laboratory of Food Science and Technology, School of Food Science and Technology, Collaborative Innovation Center of Food Safety and Quality Control, Jiangnan University, Wuxi 214122, China; 2 College of Food Science and Pharmacy, Xinjiang Agricultural University, Ürümqi 830052, China

**Keywords:** gold nanorods, metabolomics, nanotoxicity, mitochondria toxicity

## Abstract

Despite the increasing application of gold nanoparticles, there has been little assessment of biological system toxicity to evaluate their potential impact on human health. In this study, the human hepatoma cell line (Hep G2) was used in a metabolomics approach to study the effects of shape, time, and dose of gold nanorods (GNRs). Using optimized parameters for chromatography and mass spectrometry, the metabolites detected by GC-MS were processed with MS DIAL and identified with Fiehnlib. Key metabolic pathways affected by GNRs were identified by endo-metabolic profiling of cells mixed with GNRs of varying shape while varying the dose and time of exposure. The shape of GNRs affected cytotoxicity, and short GNR (GNR-S) triggered disorder of cell metabolism. High concentrations of GNRs caused more significant toxicity. The cytotoxicity and bioTEM results illustrated that the mitochondria toxicity, as the main cytotoxicity of GNRs, caused declining cytoprotective ability. The mitochondrial dysfunction disrupted alanine, aspartate, glutamate, arginine, and proline metabolism, with amino acid synthesis generally downregulated. However, the efflux function of cells can exclude GNRs extracellularly within 24 h, resulting in reduced cell mitochondrial metabolic toxicity and allowing metabolic disorders to recover to normal function.

## 1. Introduction

With increased use of engineered nanoparticles and nanomaterials in applications including consumer goods (e.g., textiles [1], cosmetics [2]) nanomedicines (e.g., drug delivery [3] and imaging agents [4] ), it is essential to characterize the fate of these materials in biological systems. However, there is insufficient information regarding the impact of manufactured nanomaterials on human health and the environment. For the successful application of nanomaterials in bioscience, it is necessary to fully understand the biological fate and potential toxicity of nanoparticles.

Gold nanorods (GNRs) have potential for use in biomedical applications, such as cancer diagnostics [5], medical treatment [6], imaging [7], and drug delivery [8], as well as applications in food [9] and beverage packaging [10] and environment remediation [11]. Although gold nanoparticles were considered nontoxic [12], recent experiments in vivo and in vitro have revealed potential health implications for use of GNRs [13]. For example, GNRs can induce cellular toxicity and hepatotoxicity in mice [14]. Additionally, GNRs can cross the blood–testis barrier (BTB) and deposit in the testes, with potential effects on male reproduction [15]. Consistent with this, human spermatotoxicity of GNRs was recently reported [16]. Preliminary cytotoxicity evaluation of gold nanomaterials of different size and shape was performed using the MTT test [17], and effects of the surface chemistry of GNRs on uptake, toxicity, and gene expression in mammalian cells were studied [18]. Changes in surface chemistry and the use of different polyelectrolytes and surfactants of varying charge can be used to manipulate the uptake of GNRs. The toxicity of cetyltrimethylammonium bromide-GNRs (CTAB-GNRs) is mainly due to CTAB residues from desorption or incomplete purification, and polyethylene glycol-GNRs (PEG-GNRs) exhibited lower toxicity than CTAB-GNRs [19,20] and showed little cytotoxicity in vitro [21].

Recent studies have utilized “omics” strategies to study either healthy or diseased biological systems. Metabolites are the products of cellular regulatory processes and have the strongest correlation to phenotype (unlike genes and proteins, whose function can be regulated by epigenetic regulation or posttranslational modification). Their levels can be viewed as the biological system’s final response to genetic or environmental changes [22]. A biological sample’s metabolites are evaluated globally using metabolomics, which measures the data that is most similar to the phenotype of the biological system being studied [23,24]. The whole complement of small-molecule metabolites present in a particular cell, organ, or organism is referred to as the metabolome [25]. Determination of the metabolome provides insights into the chemical strategies cells utilize to cope with chemical or environmental stress. The endo-metabolome (all metabolites inside the cell) and the exo-metabolome (all metabolites in the surrounding extracellular medium) make up the entire cell metabolome. Characterization of the endo-metabolome provides a more accurate picture of the cell’s metabolic behavior [26]. Therefore, the ideal method for accurately phenotyping cells, identifying crucial metabolic events underlying physiological and biochemical function, and interpreting cell responses to distinct signals, is the analysis of intracellular metabolites, despite its higher technical demands. Analyses of the endo-metabolome separately can yield complementary data [27].

Previous study revealed that low doses of GNRs could induce significant toxic effects on mitochondria and blood–testis barrier factors in TM-4 cells [15]. The present work assessed the endo-metabolome using gas chromatograph–mass spectrum (GC-MS) data to study Hep G2 cells exposed to different sizes of PEG-GNRs, including gold nanorods of long size (GNR-L), gold nanorods of short size (GNR-S), and gold nanorods of cross size (GNR-C). Cells were exposed to GNRs at different concentrations and for different lengths of time.

## 2. Materials and Methods

### 2.1. Materials and Apparatus 

The Hep G2 cell line used in the present work was obtained from the cell bank at the Chinese Academy of Sciences (Shanghai, China). Dulbecco’s modified Eagle’s medium (DMEM), fetal bovine serum (FBS), and phosphate-buffered saline (PBS) were obtained from Gibco (New York, NY, USA). Glass-bottomed dishes (35 mm) were purchased from Shengyou Biotechnology Co., Inc. (Hangzhou, China). Hydrogen tetrachloroaurate trihydrate (HAuCl4, 99%), 5-bromosalicylic acid (498.0%), hydrochloric acid (37 wt% in water), sodium borohydride (NaBH_4_, 99%), cetyltrimethylammonium bromide (CTAB), L-ascorbic acid (499.5%), thiolated PEG5000, and silver nitrate (AgNO_3_, 499%) were available commercially and were of high purity grade. Other reagents were purchased from Sinopharm Chemical Reagent Co., Ltd. (Shanghai, China). Cy5 labeled goat anti-rabbit IgG was purchased from Sangon Biotech (Shanghai, China), and the working concentration was diluted to 1:400. The phosphate-buffered solution (0.1 M K_2_HPO_4_/NaH_2_PO_4_, pH 7.4) used in this study was obtained from Sigma. All other chemicals used were of HPLC grade. Deionized water used for all experiments was purified with a Milli-Q system (Millipore, Burlington, MA, USA).

### 2.2. Cell Culture and Dosing

Hep G2 cells were cultured in a flask in DMEM medium supplemented with 10% fetal calf serum, penicillin (100 μg/mL), and streptomycin (100 μg/mL) at 37 °C in a humidified atmosphere containing 5% CO_2_ in a CO_2_ incubator (Thermo Scientific Forma Series II Water Jacket, Thermo Fisher Scientific Inc., Rockford, IL, USA). The cells were used for experiments when they reached the logarithmic growth phase after 3 days. Dosing was performed on Hep G2 cells in 12-well plates (“dosed cells”). The plates were dosed, producing a total of twelve GNR-dosed wells. In parallel, there were twelve wells in which Hep G2 cells were cultured with DMEM medium with no additional GNRs (“control cells”) or wells containing DMEM medium without Hep G2 cells that served as the “media control”. These media controls were used to determine background mass spectral data. After dissolving a predetermined quantity of GNRs in DMEM medium devoid of antibiotic or fetal calf serum, 1 mL of this solution was added to each dosed well of Hep G2 cells for 12 or 24 h.

### 2.3. Preparation and Modification of GNRs

Gold nanorods and nano-crosses were synthesized using an improved seed-mediated growth method [28]. The following method was used to prepare gold nanorods. First, NaBH_4_ was injected into the Au (III)-CTAB system while the magnetic field was vigorously stirred. To make the seed solution, the system was left at room temperature for 30 min. Second, 0.05 M CTAB was mixed with some 5-bromosalicylic acid, and AgNO_3_, 1 mM HAuCl_4_ solution, and a small amount of HCl were added under slow magnetic stirring for 15 min. Stirring was stopped when the solution became colorless, and then 0.064 M ascorbic acid was added. In the end, the growth solution was injected with the prepared seed solution. After 30 s of stirring, the mixture was left to stand at room temperature for 12 h. Gold nano-crosses were prepared similarly by substituting 5-bromosalicylic acid for sodium salicylate [29].

In order to obtain GNR-PEG, thiolated PEG5000 was used to modify the facets of GNRs at a PEG/GNRs molar ratio of 30:1. To do this, a 1 mL aliquot of concentrated GNRs was mixed with 0.4 mL ultrapure water, then 0.6 mL of 5 mM PEG was added under vigorous stirring. After incubation for 12 h, the excess PEG was removed by centrifugation at 14,000 rpm for 10 min and then this material was dissolved in 1 mL of 5 mM CTAB solution.

For Cy5-labeled GNRs, the procedure was as follows. First, the disulfide bonds in Cy5-labeled ssDNA were reduced by TCEP. Then, the reduced ssDNA-Cy5 was added to the corresponding Au solution to make the Au–thiol interaction to obtain Cy5-modified GNRs.

Measurement of particle size and concentration of GNRs: The particle concentration was measured by UV–vis spectroscopy using molar extinction coefficients at the wavelength of maximum absorption of each gold colloid, as reported recently [17]. Specifically, 1 mL of GNRs was taken in a cuvette and scanned for absorbance in the 400–1000 nm interval. The particle size of GNRs was determined by dynamic light scattering (DLS). The sample concentration was in the range of 0.001–1 mg/mL or particle density between 10^9^–10^12^ particles/mL. The concentration could be adjusted appropriately so that the scattered light intensity suitable for the sample met the instrumental detection requirements.

### 2.4. Metabolite Extraction and GC-MS Analysis

On ice, the cell dishes were laid out, and 1 mL of chilled water was applied three times. In addition to the dosed cells and control cells, we also measured a blank sample to account for the background signals. Next, 1 mL of chilled methanol/H_2_O (3:2, *v*/*v*) quenching solvent was added to each plate and cells were scraped with a cell lifter, transferred to 1.5 mL Eppendorf tubes, and centrifuged at 4 °C for 5 min at 1000 g. After discarding the supernatant, the cell precipitate was stored at −80 °C or analyzed immediately. Ice-cold extraction solution (0.5 mL of acetonitrile/isopropanol/H_2_O (3:3:2, *v*/*v*/*v*)) and two stainless steel beads were added into the tubes. Next, cells were subjected to grinding using a grinder at 1500 rpm for five cycles of 30 s. After the homogenate was centrifuged, the supernatant was transferred into a new tube. This procedure was repeated for the precipitate. The supernatant was then divided into two 480 μL aliquots, with one used for analysis and the other kept as a backup. Finally, the samples were freeze-dried and stored at −20 °C for analysis. For quality control, equal volumes were taken from all samples and combined for use as a mixed sample.

### 2.5. Derivatization of GC-MS Samples

Methoxylamine hydrochloride was added into dried samples and allowed to incubate at 30 °C for 90 min. Next, N-methyl-N-(trimethylsily)ltrifluoroacetamide (MSTFA) with 1% trimethylchlorosilane was added into each sample, followed by incubation at 37 °C for 30 min. As an internal standard, we used 1 mg/mL fatty acid/methyl ester mixture (FAMEs, C8–C16: 1 mg/mL; C18–C24: 0.5 mg/mL in chloroform). The derivatized samples were analyzed by GC-MS within 24 h.

For GC-MS analysis, a Shimadzu QP2010 Ultra gas chromatograph coupled with a mass spectrometer was used with a Rxi-5 Sil MS column (30 m × 250 μm inner diameter, 0.25 μm film thickness; Restek, Bellefonte, PA, USA). Helium was used as the carrier gas. The column gas flow rate was 20 mL min^−1^ and the front inlet purge flow was 5 mL min^−1^. 1 µL of sample was injected in a splitless mode. The initial temperature of the column was 50 °C for 0.5 min, and the temperature was then raised to 110 °C at a rate of 30 °C min^−1^, increased to 310 °C at the rate of 10 °C min^−1^, and then maintained at this temperature for 10 min. The temperatures of the injection, transfer line, and ion source were 280, 280, and 250 °C, respectively. The EI voltage was −70 eV in electron impact mode. The mass spectrometry data were acquired in full-scan mode with a *m*/*z* range of 85–500 at a rate of 17 spectra per second with a solvent delay of 240 s.

### 2.6. Metabolite Profiling Analysis

The raw data were converted using GC-MS PostRun from Shimazu and the ABF converter software (http://www.reifycs.com/AbfConverter/index.html, accessed on 15 November 2019), and the final format was “abf”. The MS DIAL software (http://prime.psc.riken.jp/compms/msdial/main.html, accessed on 15 November 2019) [30] with Fiehnlib [31] was used for peak extraction, data baseline filtering, calibration of the baseline, peak alignment, deconvolution analysis, peak identification, and integration of the peak height.

### 2.7. Data Analysis

Statistical analysis was performed with R. The results were analyzed by *t*-test. Data were expressed as mean values ± standard deviation (SD). Significance was set at *p* < 0.05. In this experiment, Metaboanalyst 4.0 (https://www.metaboanalyst.ca/, accessed on 15 November 2019) was used to perform integrating enrichment analysis [32]. R was used for the principal component analysis (PCA) and heatmap analysis. Pathway mapping was performed with MetaMapp (http://metamapp.fiehnlab.ucdavis.edu/ocpu/library/MetaMapp2020/www/, accessed on 15 November 2019) [33] and visualized by CytoScape 3.4.0 (https://cytoscape.org/, accessed on 15 November 2019) [34].

## 3. Results and Discussion

### 3.1. Characterization of GNRs and Cell Toxicity Test

**Figure 1:**
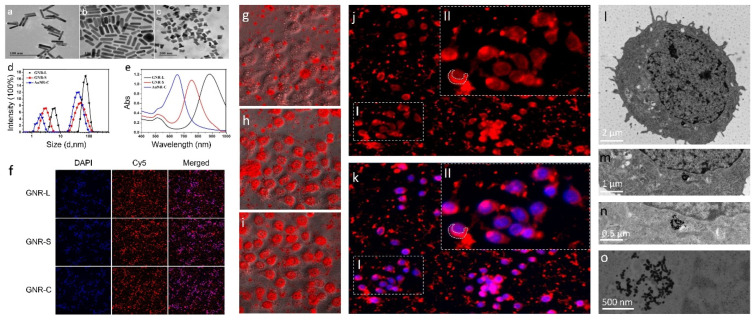
The transmission electron microscope imaging of the three kinds of GNRs: (**a**) GNR-L, (**b**) GNR-S, (**c**) GNR-C. (**d**) Particle size analysis of the three kinds of GNRs. (**e**) UV analysis of the three kinds of GNRs. Representative images from the HCS after GNR (GNR-L, GNR-S, GNR-C) exposure to Hep G2 cells for 12 h. (**f**) Cell nucleus (blue) and GNRs (red). Images were acquired with an High Content screening (HCS). HCS images of Hep G2 cells treated with GNR-L for multiple time points, (**g**) 0 h, (**h**) 12 h, (**i**) 24 h. Confocal images of Hep G2 cells treated with GNR-L for 12 h, (**j**) Cy5-labeled GNR-L, (**k**) Cy5-labeled GNR-L and DAPI labeled Hep G2 cells. Part II in the figure is the enlarged result of part I in the figure. Biological transmission electron microscope imaging of Hep G2 cells treated with GNRs, (**l**) 2 μm, (**m**) 1 μm, (**n**) 0.5 μm, (**o**) 500 nm.

The transmission electron microscopy (TEM) images of different shapes of GNRs are shown in Figure 1a–c, revealing the expected different sizes of GNRs. More TME images of GNRs are shown in Figure S1. The lengths of GNR-Long (Figure 1a), GNR-Short (Figure 1b), and GNR-Cross (Figure 1c) particles were approximately 70 nm, 35 nm, and 20 nm, respectively. The widths of GNR-Long, GNR-Short, and GNR-Cross particles were approximately 8 nm, 8 nm, and 10 nm, respectively.

To further confirm the size of the three synthetized GNRs of different shapes, dynamic light scattering (DLS) was used. As shown in Figure 1d, the DLS revealed that all the GNRs exhibited narrow size distributions, with average diameters of 70 nm, 38 nm, and 28 nm for the GNR-L, GNR-S, and GNR-C particles respectively. The aspect ratios of the three GNR particle shapes were 7, 3.5, and 2, respectively. The DLS diameters were measured for GNRs modified with or without PEG, as shown in Figure S2. The GNRs exhibited longitudinal surface plasmon resonance (LSPR) absorbance at 880 nm, 785 nm, and 665 nm, and transverse surface plasmon resonance (TSPR) peak at 511 nm, as shown in Figure 1e. GNRs, modified with red fluorescein, Cy5, whose excitation wavelength is 646 nm and emission wavelength is 662 nm, were incubated with Hep G2 cells at 37 °C for 12 h. As shown in Figure 1f, GNRs with different shapes could enter the Hep G2 cell membrane in 12 h. To optimize the incubation time of GNRs with cells, GNR-L marked with Cy5 was mixed with Hep G2 cells and subjected to real-time monitoring for 24 h. In the first six hours, the GNR-L appeared randomly dispersed in the Hep G2 cell dish by gravity action, as shown in Figure 1g. It could be observed plainly in magnified figures that the GNR-L had not yet penetrated into the Hep G2 cells. After incubating for 12 h, the GNR-L were rearranged according to the cell shape, which demonstrated that the GNR-L had entered the Hep G2 cells (Figure 1h). With increased time, no additional obvious difference was observed (Figure 1i).

The washed Hep G2 cells were marked with diamidino-phenyl-indolent (DAPI) and analyzed through Confocal. In Figure 1j,k, the purple fluorescence in merged figures illustrates that the GNRs marked with red fluorescence signals entered the Hep G2 cells, whose cellular nuclei are marked with blue fluorescence. The cytotoxicity of the GNRs in different cell lines was tested using a cholecystokinin (CCK-8) kit and the results are presented in Figure S3. The Hep G2 cells (hepatotoxicity) were selected for toxicity evaluation of GNRs because they are more sensitive to GNRs. As shown in the zoomed image in Figure 1(kII), the Cy5-labeled GNR-L were located mainly in the cytoplasm. This conclusion was confirmed by biological transmission electron microscope (BTEM) imaging for Hep G2 cells treated with three kinds of GNRs for 12 h, as shown in Figure 1l–o. Additional bio-transmission electron micrographs are shown in Figures S4 and S5. The Hep G2 cells were dehydrated, fixed, histologically sectioned, and then imaged by BTEM. All three kinds of GNRs were observed in BTEM figures. No GNRs were observed at the cell membrane, with most GNRs localized in the cytoplasm, particularly the mitochondria and endoplasmic network [35], similar to previous reports. The penetration process of GNRs can be graphically presented in three stages. At Stage I, GNR particles were distributed by gravity in the culture medium in a random scattered pattern. At Stage II, GNR particles were gradually engulfed by cells and became discretely distributed in the cell membrane and the endosomal–lysosomal system. At Stage III, GNR particles aggregated and were mainly located in the mitochondria and possibly other subcellular organelles. The GNRs are proposed to cause some agglutination in mitochondria [36]. GNRs ruptured the endosomal/lysosomal membrane, resulting in the release of nanorods and affecting the mitochondria. Nano-scale particles may be able to enter the blood tissue and cells, but this has not been observed in vitro. An additional issue is potential cytotoxicity induced by the permanent residual nanosomes [37].

Real-time monitoring was performed using HCS to record the entry of GNRs into cells. HCS automatically took one photo each hour with no manual action required for extra lens focus or exposure time setting. The time series monitoring result is shown in Figure S6, where it can be observed that GNRs randomly dispersed in the Hep G2 cell dish by gravity action in the first 6 h. With incubation time increasing to 12 h, the GNRs entered the cells, with no additional significant change after 12 h. Figure S7 shows a flow diagram that briefly describes the distribution stages of GNRs in cells. Intuitively, the rearrangement of GNRs according to the cell shape demonstrated that GNRs had accessed the cell. Hep G2 cells were washed with PBS three times to remove background fluorescence contamination before measurement of the intracellular distribution of GNRs.

### 3.2. GNR Shape Effect on Hep G2 Cell Metabolism

GNRs were speculated to undergo various agglutination processes in mitochondria [38], or to rupture the endosomal membrane [39] and lysosomal membrane [40], inducing cell metabolism disorders [41]. With their nano-scale body size, GNRs could easily cross into the blood tissue, or enter cells, and GNR toxicity was variable based on exposed cell type and dependent on the GNRs’ composition and size [42]. Permanent residues of GNRs could have cytotoxicity or lead to some metabolic function disorders. Mitochondria are significant organelles in cells, and functional deficiency could result in serious diseases. To better understand the potential cellular cytotoxicity of GNRs in cell metabolism, endo-metabolism analysis was performed with GC/MS-based metabolomics.

**Figure 2:**
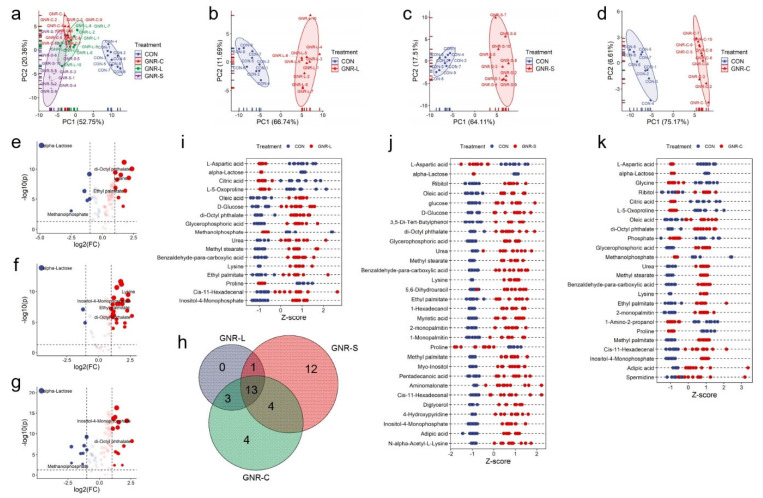
(**a**) PCA of endo-metabolites of Hep G2 cells treated with different shaped GNRs (GNR-L, GNR-S, and GNR-C). PCA of endo-metabolites of Hep G2 cells treated with (**b**) GNR-L, (**c**) GNR-S, and (**d**) GNR-C for 12 h. Volcano plots of endo-metabolites of Hep G2 cells treated with (**e**) GNR-L, (**f**) GNR-S, and (**g**) GNR-C for 12 h. (**h**) Venn diagrams of statistically significant changed metabolites of Hep G2 cells treated with GNR-L, GNR-S, and GNR-C. Z-score plot of statistically significant changed metabolites of Hep G2 cells treated with (**i**) GNR-L, (**j**) GNR-S, (**k**) GNR-C. The metabolites were defined as showing statistically significant change if *p* value was <0.05, and fold change value was >2 or <0.5.

The endo-metabolites of Hep G2 cells treated with GNRs of different shapes (GNR-L, GNR-S, GNR-C) were determined and the results of multivariate statistical analysis and a principal component analysis (PCA) scores plot are shown in Figure 2a, revealing the separation of clusters of different metabolite groups. Noticeable differences between the groups dosed with GNRs and control groups were observed, indicating that the GNRs altered metabolic function in Hep G2 cells. There was little variation in the metabolites of the three groups treated with GNRs of different shapes, indicating that shape does not have a significant effect. To delve into the differences in cellular metabolism of GNRs with different shapes, volcano plots were constructed to identify the metabolites with statistically significant differences. As shown in Figure 2e–g, more metabolites were upregulated in the GNR-S group, leading to the different PCA score in Figure 2a. A Venn diagram was constructed to illustrate the composition of metabolites with statistically significant differences, as shown in Figure 2h, and this indicates that 13 metabolites exhibited intersectional relationships between the three GNR groups. However, 12 metabolites showed differences only in the GNR-S group, indicating that GNR-S caused additional effects on Hep G2 cells. This may have been due to the smaller volume of GNR-S, which could facilitate penetration of the cells’ protective barrier to produce other toxic effects [43]. Z-score plots were constructed to display the statistically significant metabolites in the three GNR-dosed groups, as shown in Figure 2i–k. Some metabolites displayed the same decreasing tendency, with such effects seen in L-aspartic acid, alpha-lactose, and proline, while other metabolites displayed the opposite pattern, with such effects seen in urea and lysine. We concluded that GNR-S leads to more severe metabolic disorder, with greater upregulation of metabolites such as myo-inositol, 4-hydroxypridine, and N-alpha-acetyl-L-lysine, which only significantly changed in GNR-S-dosed Hep G2 cells.

### 3.3. Concentration Effect of GNRs on Hep G2 Cell Metabolism

The concentration of GNRs was indirectly calculated based on the intensity of LSPR absorption (Abs), considering the molar extinction coefficients (ξ), related to the aspect ratio of GNRs and the thickness of the quartz cell (d). The concentration of GNRs (C_GNRs_) was calculated using the following formula [44]:

Abs = ξ (cm^−1^·M^−1^) × d (cm) × C_GNRs_ (M)

**Figure 3:**
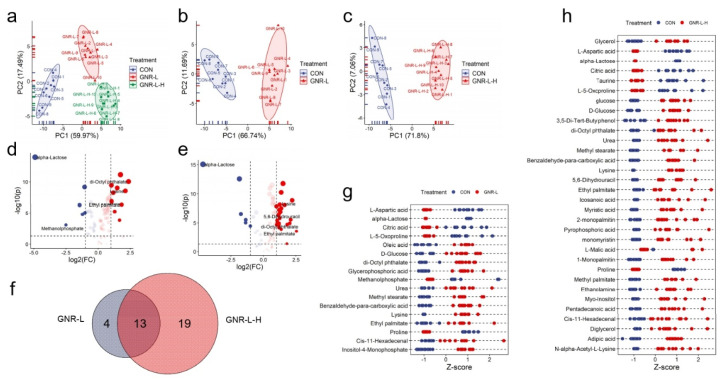
(**a**) PCA of endo-metabolites of Hep G2 cells treated with GNR-L of different concentrations (GNR-L, GNR-L-H). PCA of endo-metabolites of Hep G2 cells treated with (**b**) GNR-L and (**c**) GNR-L-H for 12 h. Volcano plot of endo-metabolites of Hep G2 cells treated with (**d**) GNR-L and (**e**) GNR-L-H. (**f**) Venn diagram of statistically significant changed metabolites of Hep G2 cells treated with GNR-L and GNR-L-H. Z-score plots of statistically significant changed metabolites of Hep G2 cells treated with (**g**) GNR-L and (**h**) GNR-L-H. The statistically significant changed metabolites were considered significant if *p* value was <0.05 and fold change value was >2 or <0.5.

The viability of Hep G2 cells (hepatotoxicity) was tested for treatment with GNRs of concentration: 0.02, 0.025, 0.03, 0.035, 0.04, 0.045, and 0.05 Abs (Figure S3). The GNR-L represents a concentration of 0.02 Abs (3.5 pM, ξ = 5.5 × 10^9^), and GNR-L-H represents a concentration of 0.025 Abs (4.5 pM, ξ = 5.5 × 10^9^). As shown in Figure 3, the hypothesis matched the metabolomics result that high concentration triggered more serious metabolic disorders, showing greater upregulation of metabolites. The PCA scores of GNR-L and GNR-L-H illustrated cluster differences compared with the control group (Figure 3a–c). It could be seen in the volcano plots that more metabolites showed statistically significant differences in the GNR-L-H-dosed group (Figure 3d,e), which indicated that higher concentration of GNR-L resulted in more obvious effects on the metabolic phenotype of Hep G2 cells. A Venn diagram was constructed and shows the quantitative analysis of differences in statistically significant metabolites between the two groups, shown in Figure 3f, revealing 13 metabolites with significant changes in both groups. An additional 19 metabolites only changed in the GNR-L-H group. The changed metabolites are displayed in Z-score plots in Figure 3g,h, which more intuitively show the significant metabolite differences in high-dose and low-dose GNR-treated groups.

### 3.4. Time of Exposure Effect on Hep G2 Cell Metabolism

**Figure 4:**
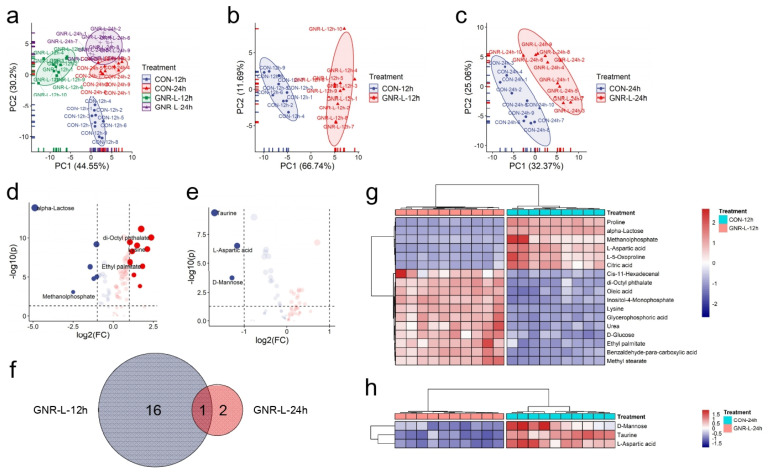
(**a**) PCA of endo-metabolites of Hep G2 cells treated with GNR-L for different amounts of time (GNR-L-12 h, GNR-L-24 h). PCA of endo-metabolites of Hep G2 cells for (**b**) GNR-L-12 h and (**c**) GNR-L-24 h. Volcano plots of endo-metabolites of Hep G2 cells treated with (**d**) GNR-L-12 h and (**e**) GNR-L-24 h. (**f**) Venn diagram of statistically significant changed metabolites of Hep G2 cells for GNR-L-12 h and GNR-L-24 h groups. Heatmap of statistically significant changed metabolites of Hep G2 cells treated with (**g**) GNR-L-12 h and (**h**) GNR-L-24 h. The statistically significant changed metabolites were selected if *p* value was <0.05 and fold change value was >2 or <0.5.

We next investigated the effect of exposure time on cellular metabolism induced by GNRs as a potential determinant of cytotoxicity of GNRs. PCA scores indicated that both GNR-L-12 h and GNR-L-24 h groups differed in principal components compared with the control group (12 h and 24 h). A slight difference in the principal components was seen for the control groups measured at 12 h and 24 h, as shown in Figure 4a. Figure 4b,c shows a longer Mahalanobis distance of GNR-L-12 h than that of GNR-L-24 h, which indicated less change in the principal components of GNR-L-24 h. The volcano plots in Figure 4d,e display the statistically significant changes in metabolites of the GNR-L-12 h group and GNR-L-24 h group. A total of 17 metabolites showed statistical differences in the GNR-L-12 h group, while in the GNR-L-24 h group there were only three metabolites that showed statistical differences, and only one metabolite, L-aspartic acid, was shared between these groups, as shown in Figure 4f. Heatmap analysis was used to display themetabolites with statistical differences in the GNR-L-12 h and GNR-L-24 h groups, as shown in Figure 4g,h. Significant metabolic differences occurred at 12 h, and this difference was gradually reduced at 24 h, which was surprising. Cells may respond to stress caused by GNRs in a short period of time, resulting in metabolic disorders. After a certain period of time, however, the cells may effectively repair or protect the cell body to reduce the toxicity of the GNRs [45]. Another possibility is that cells may be able to pump GNRs out of the cell in some way after the stress reaction. Therefore, it is important to study the extracellular metabolism of cells, and explore the metabolic effects of GNRs on cells and the mechanism of cell self-protection.

### 3.5. Pathway Analysis of Hep G2 Cells Dosed with GNR

**Figure 5:**
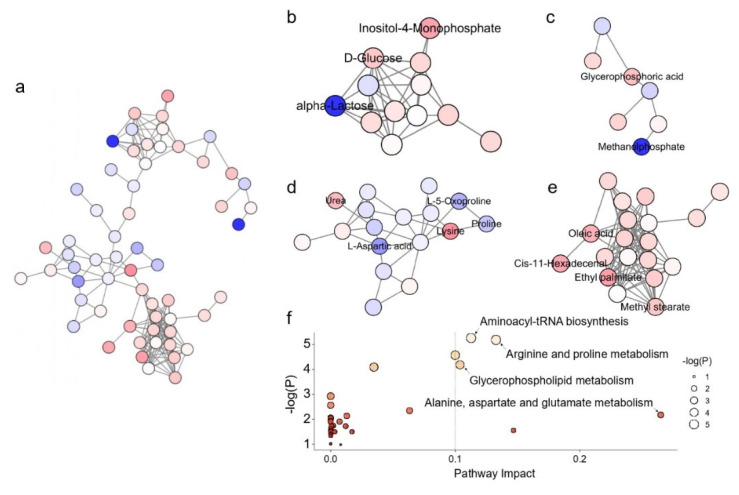
Metabolic network analysis of Hep G2 cells treated with GNR-L for 12 h: (**a**) all significant changed metabolites, (**b**) carbohydrate-related metabolites, (**c**) phosphorylate-related metabolites, (**d**) amino acid-related metabolites, (**e**) fatty acid-related metabolites. (**f**) Pathway enrichment analysis of significantly changed metabolites. Significantly changed pathways based on enrichment and topology analysis are shown. The *x*-axis represents pathway enrichment, and the *y*-axis represents pathway impact. Large sizes and yellow colors represent major pathway enrichment and high pathway impact values, respectively.

The associated metabolic pathways were created by MetaMapp and visualized using CytoScape. According to the fold change direction estimated by MetaMapp, the red color denotes increased metabolites in comparison to the control group, the blue color denotes decreased metabolites, and the white color denotes metabolites with insignificant change. Lines between two metabolites indicate the connected metabolites share a similar functional group or similar chemical structure, as defined by PubChem. Figure 5a shows the metabolic pathway analysis of the Hep G2 cellular metabolic network with identified metabolites after treatment with GNR-L for 12 h, as generated by MetaMapp, and visualized by CytoScape. To better visualize the metabolic pathway, the metabolites were arranged in the organic layout. It can be clearly seen in Figure 5a that metabolites of different classes, such as carbohydrate-related metabolites, phosphorylate-related metabolites, amino acid-related metabolites, and fatty acid-related metabolites, clustered separately. A boxplot of significantly changed metabolites in the 12 h GNR-L-dosed group is shown in Figure S8. As shown in Figure 5b, the carbohydrate-related metabolic network, inositol-4-monophosphate, and D-glucose significantly increased when Hep G2 cells were treated with GNR-L, while alpha-lactose significantly decreased. Glucose arises from the breakdown of glycogen in a process known as glycogenolysis. GNR-L could promote metabolic disorder in glycogenolysis and inositol phosphate metabolism of Hep G2 cells. The strongly significant decrease of alpha-lactose indicates metabolic abnormality in lactose synthesis pathway. Lactose downregulation may cause cells to activate glycogenolysis to produce more glucose for energy. Energy metabolism is mainly related to the function of mitochondria. Programmed cell death may result from mitochondrial perturbation, and accumulated GNRs in mitochondria may be responsible for cellular damage. As shown in Figure 5c, of the phosphorylate-related metabolites, glycerophosphoric acid significantly increased and methanolphosphate significantly decreased. Glycerophosphoric acid is a component of glycerolipid and glycerophospholid metabolism, and is closely linked to fatty acid metabolism. Glycerophospholid metabolic disorder induces a biphasic burst of superoxide anions and regulates MAPK (mitogen-activated protein kinases)-mediated apoptosis [46]. Increasing production of phosphatidic acid on the mitochondrial surface results in mitochondrial aggregation and facilitates the fusion process [47], which can affect the lysosomal–mitochondrial mediated apoptotic pathway [48]. As shown in Figure 5e, all the annotated fatty acid-related metabolites showed an upward trend, including oleic acid, cis-11-hexadecenal, ethyl palmitate, and methyl stearate. The changes in fatty acid metabolism, such as levels of ethyl palmitate and oleic acid, will correspond with changes in lipid metabolism, which is regulated by multiple signaling pathways and generates a variety of bioactive lipid molecules. As shown in Figure 5d, for amino acid-related metabolism, most amino acids showed a downward trend, including L-5-oxoproline, proline, and L-aspartic acid, but lysine increased. In the urinary circulation metabolism, urea showed an upward trend. The slightly intensified downregulation of L-5-oxoproline, proline, and L-aspartic acid suggested increased mitochondria toxicity of GNRs with increased incubation time. Of these changed metabolites, 5-oxoproline is a cyclized derivative of glutamic acid, and proline is a non-essential amino acid that is synthesized from glutamic acid, so both are related to alanine, aspartate, and glutamate metabolism, and arginine and proline metabolism. The downregulated L-aspartic acid may indicate citrate cycle metabolic abnormality.

The statistically significantly changed metabolites were subjected to metabolic pathway enrichment analysis using the web-based MetaboAnalyst 4.0 tool. The results are shown in Figure 5f, and indicated the main effects on alanine, aspartate, glutamate, arginine, proline, and glycerophospholipid metabolism, which were consistent with the metabolic network analysis results.

## 4. Conclusions

The purpose of this study was to examine cell-dependent responses to GNRs of variable shapes and sizes through monitoring their cellular uptake, cell viability, intracellular responses and potential mechanisms of toxicity. To distinguish these effects, the cell culture model was selected to evaluate a range of concentrations of GNRs to additionally investigate concentration-dependent effects. To delve into the differences in cellular metabolism of GMRs with different shapes, a GC–MS-based metabolomics platform was utilized for study of the mechanisms of metabolic toxicity. The results indicated that GNR-S triggered more serious disorder of cell metabolism, and high concentrations of GNRs caused more significant toxicity. The main toxicity mechanism of GNRs on Hep G2 cell lines was the accumulation of GNRs in cell sub-organs, such as mitochondria, affecting TCA cyclic metabolism and thereby reducing energy production. Cells have to activate glycogenolysis and related metabolic pathways, such as glycerolipid and glycerophospholid metabolism, to supply enough energy. Mitochondrial dysfunction affects alanine, aspartate, glutamate, arginine, and proline metabolism, with additional downregulation of amino acids including L-5-oxoproline, proline, and L-aspartic acid. However, cells may be able to expel GNRs by pumping or some other form of efflux to reduce the quantity of GNRs in the cells, thereby reducing continuous toxicity.

## Data Availability

All related data and methods are presented in this paper. Additional inquiries should be addressed to the corresponding author.

## Supplementary Materials

The following supporting information can be downloaded at: https://www.mdpi.com/article/10.3390/foods11223569/s1, Figure S1: The DLS diameter and UV–vis spectra of GNRs modified with or without PEG; Figure S2: The transmission electron microscopy (TEM) image of various sizes based GNRs; Figure S3: The cytotoxicity test of the GNRs in three different kinds of cell lines; Figure S4: Transmission electron micrographs show the Hep G2 cells with GNRs; Figure S5: Bio-transmission electron micrographs show the Hep G2 cells with GNRs; Figure S6: The HCS images of Hep G2 cell incubated with GNRs modified with Cy-5 fluorescent probe; Figure S7: The HCS images of Hep G2 cell incubated with GNRs modified with Cy-5 under larger magnification scale; Figure S8: Boxplot of significant changed metabolites in GNR-L-dosed group for 12 h.
