# Supplementary material for: Cell-Based Metabolomics Approach for Anticipating and Investigating Cytotoxicity of Gold Nanorods
Source: Foods. 2022 Nov 9;11(22):3569. doi: 10.3390/foods11223569 (PMC9689499; doi:10.3390/foods11223569)
Supplement: Supplementary file 1 [file foods-11-03569-s001.zip › foods-1976735-supplementary.pdf]

Supporting figures

Figures:

Figure S1. The DLS diameter and UV-vis spectra of GNRs modified with or without PEG.

Figure S2. The transmission electron microscopy (TEM) image of various sizes based GNRs.

Figure S3. The cytotoxicity test of the GNRs in three different kinds of cell lines

Figure S4. Transmission electron micrographs show the Hep G2 cells with GNRs

Figure S5. Bio-Transmission electron micrographs show the Hep G2 cells with GNRs

Figure S6. The HCS images of Hep G2 cell incubated with GNRs modified with Cy-5 fluorescent probe

Figure S7. The HCS images of Hep G2 cell incubated with GNRs modified with Cy-5 under larger magnification scale

Figure S8. Boxplot of significant changed metabolites in GNR-L dosed group for 12 h

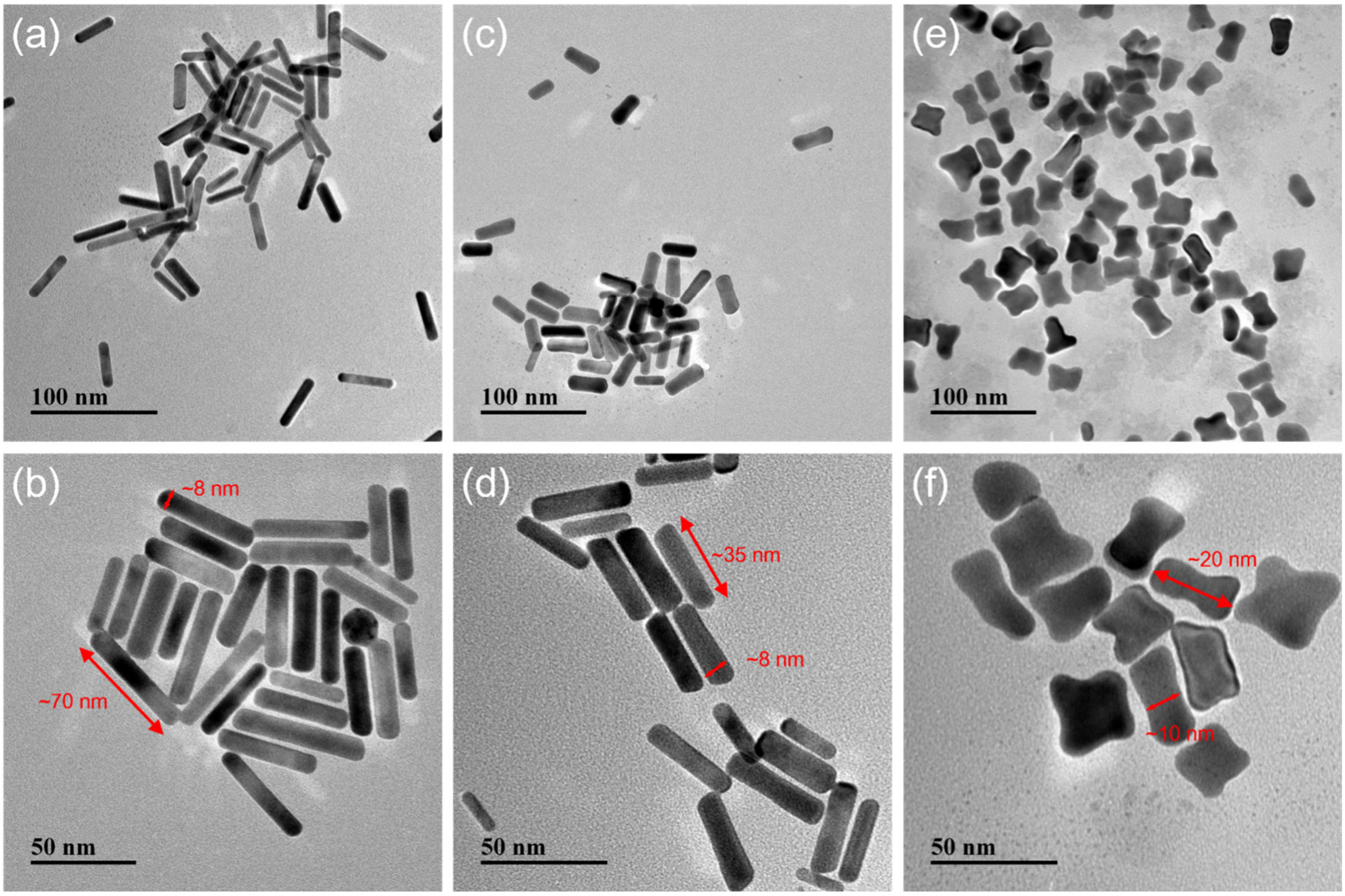

Figure S1. The transmission electron microscopy (TEM) image of GNRs. (a) The TEM image of GNR-L and (b) the zoomed TEM image of GNR-L. The length and width of GNR-L were approximate 70 nm and 8 nm. (c) The TEM image of GNR-S and (d) the zoomed TEM image of GNR-S. The length and width of GNR-S were approximate 35 nm and 8 nm. (e) The TEM image of GNR-S and (f) the zoomed TEM image of GNR-S. The length and width of GNR-C were approximate 20 nm and 10 nm. No much visible difference between GNRs and GNRs modified with PEG was observed in TEM image.

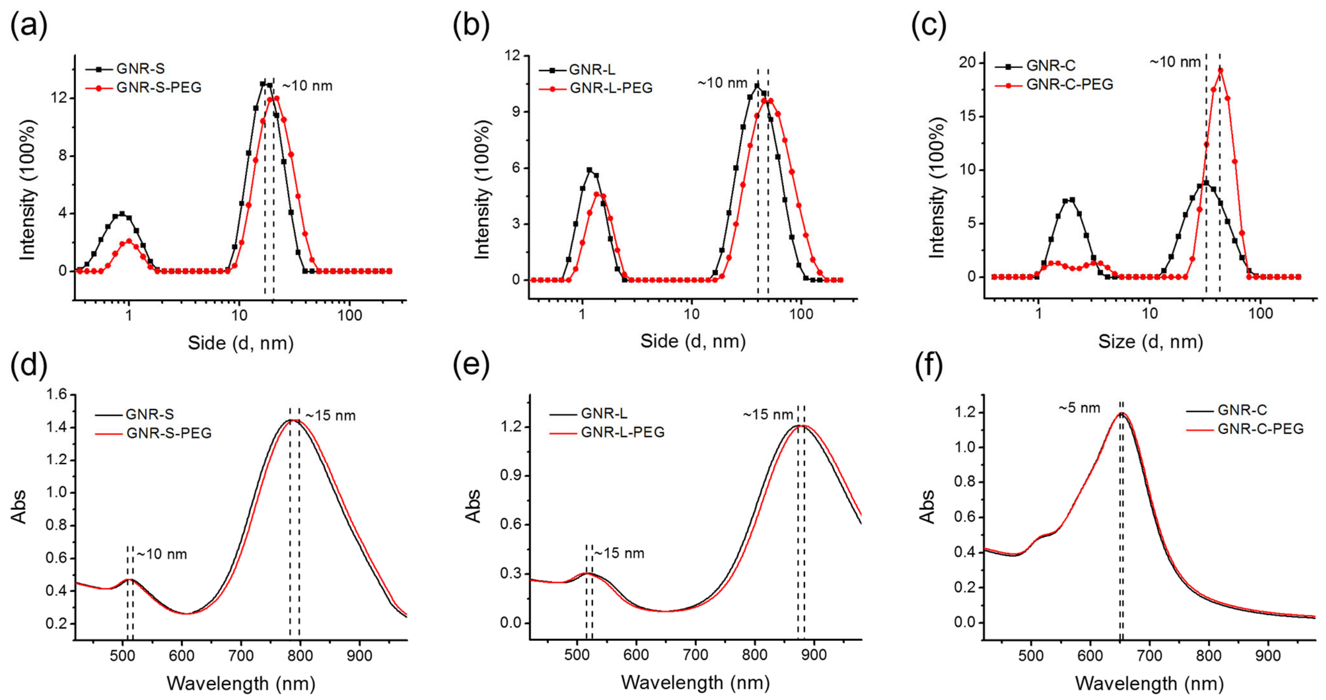

Figure S2. The dynamic light scattering (DLS) diameter of various sizes based GNRs modified with or without PEG. (a) DLS diameter of GNR-S and GNR-S-PEG, with approximate 10 nm size distinction. (b) DLS diameter of GNR-L and GNR-L-PEG, with approximate 10 nm size distinction. (c) DLS diameter of GNR-C and GNR-C-PEG, with approximate 10 nm size distinction. The UV-vis spectra of various sizes based GNRs modified with or without PEG. (d) Longitudinal surface plasmon resonance (LSPR) and transverse surface plasmon resonance (TSPR) of GNR-S were at 785 nm and 511 nm, with about 10 nm difference from GNR-S-PEG, (e) LSPR and TSPR of GNR-L were at 880 nm and 511 nm, with about 15 nm difference from GNR-L-PEG. (f) LSPR and TSPR of GNR-C were at 665nm and 511 nm, with about 5 nm difference from GNR-C-PEG.

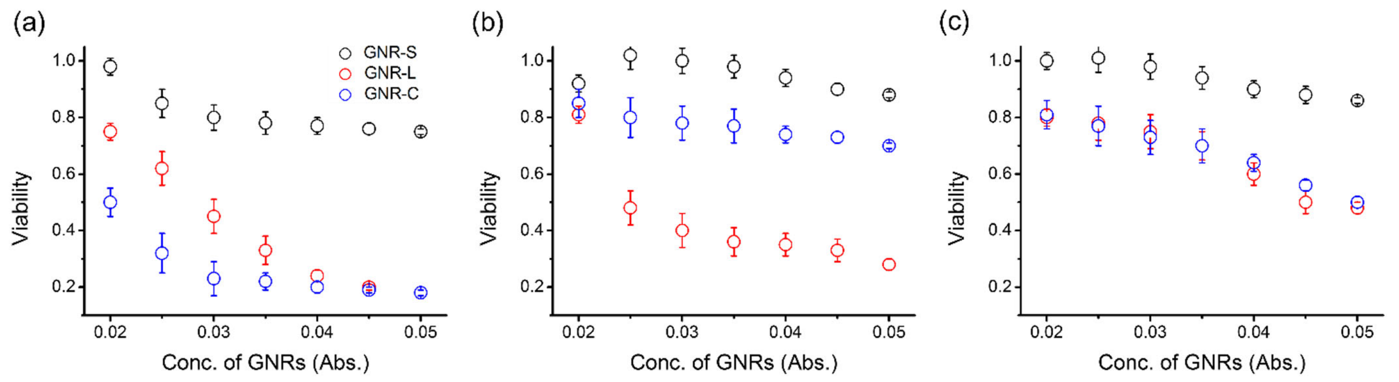

Figure S3. The cytotoxicity test of the GNRs in three different kinds of cell lines by cholecystokinin (CCK-8) kit. (a) The viability test of Hep G2 cells (hepatotoxicity) treated with GNRs of concentration: 0.02, 0.025, 0.03, 0.035, 0.04, 0.045 and 0.05 Abs. (b) The viability test of HEK293 cells (nephrotoxicity) treated with GNRs of concentration: 0.02, 0.025, 0.03, 0.035, 0.04, 0.045 and 0.05 Abs. (c) The viability test of ANA-1 cells (immunotoxicity) treated with GNRs of concentration: 0.02, 0.025, 0.03, 0.035, 0.04, 0.045 and 0.05 Abs

Concentration calculation:

$\text{Abs} = \xi(\text{cm}^{-1}\ \text{M}^{-1}) \times \text{d}(\text{cm}^{-1}) \times \text{C(M)}$

Abs: the intensity of longitudinal surface plasmon resonance absorption.

ξ: molar extinction coefficients, based on the aspect ratio of GNRs[2].

d: the thickness of quartz cell

C: the concentration of GNRs

Three kinds of main cell lines, including Hep G2, HEK 293, and Ana 1 cell, were used for preliminary cytotoxicity test of different shapes of GNRs with various concentrations. The comparison result was accessible in Fig. S1. Shapes was not the only factor on the cellular toxicity of GNRs. The viability of the three cell lines inhibited different sensitiveness to different shapes of GNRs. However, the obviously observation could be made that the GNRs-Long (GNRs-L) had definitely high cytotoxicity on the three types of cells, and GNRs-Short (GNRs-S) had a slighter cytotoxicity comparing with the GNRs-Cross (GNRs-C). This brief conclusion agreed with the previous opinion that obvious differences in cellular uptake, intracellular trafficking, and susceptibility of lysosome to GNRs by different types of cell lines resulted in selective accumulation of GNRs in the mitochondria. To better understand the metabolism toxicity of GNRs, the Hep G2 cell line, was selected as model cell, as liver plays a major role in metabolism with numerous functions, particularly for glycogenolysis and detoxification. And the optimized dose of GNRs for cell metabolism study was that induced around 20% cells dead.

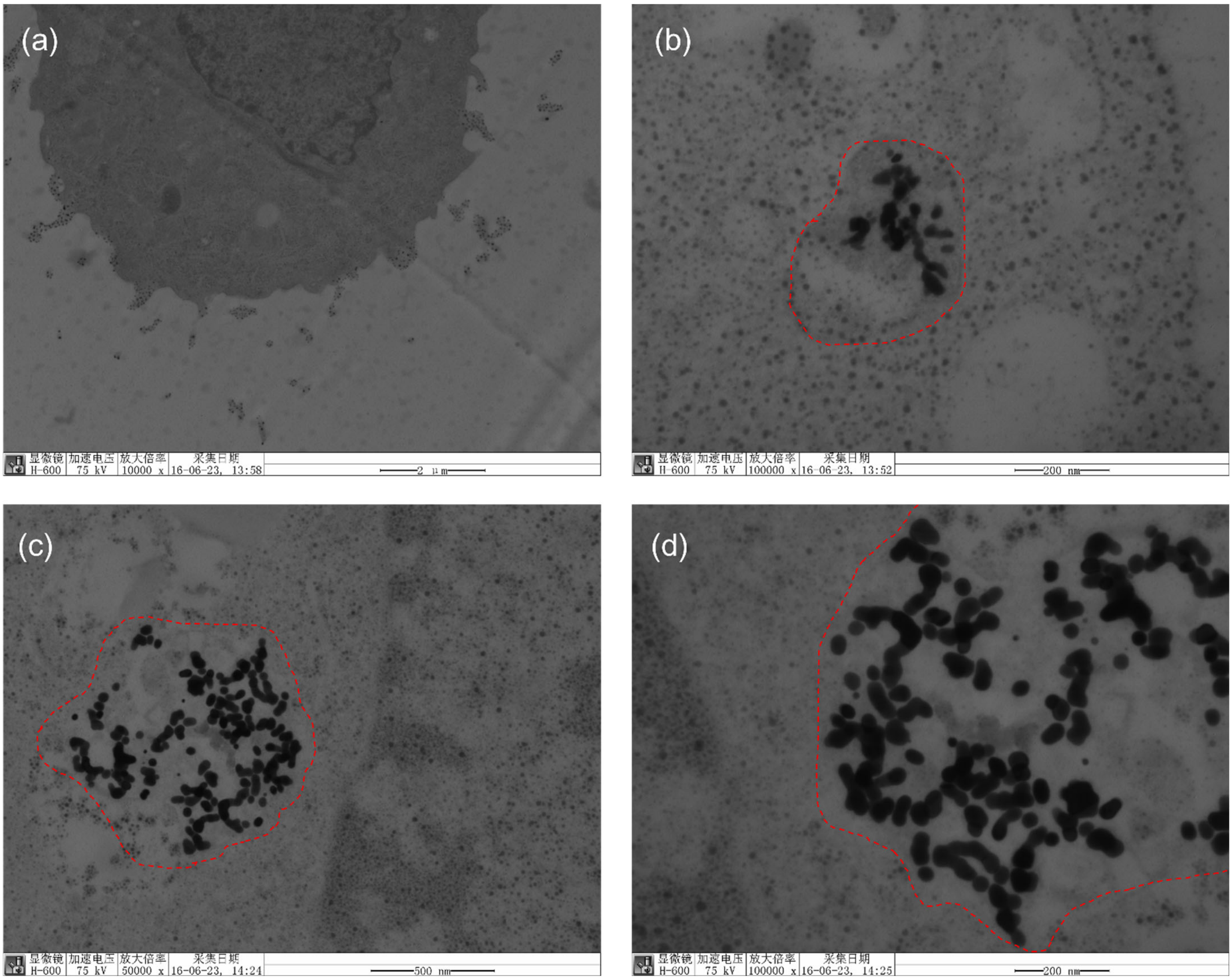

Figure S4. Electron micrographs at different magnifications of a cell containing nanoparticles. Cells were exposed to nanoparticles for 12h, fixed with osmium tetroxide, sectioned, and visualized with a Hitachi H-8000 electron microscope. (a) zoom rate 10000 x; (b) zoom rate 100000 x; (c) zoom rate 50000 x; (d) zoom rate 100000 x.

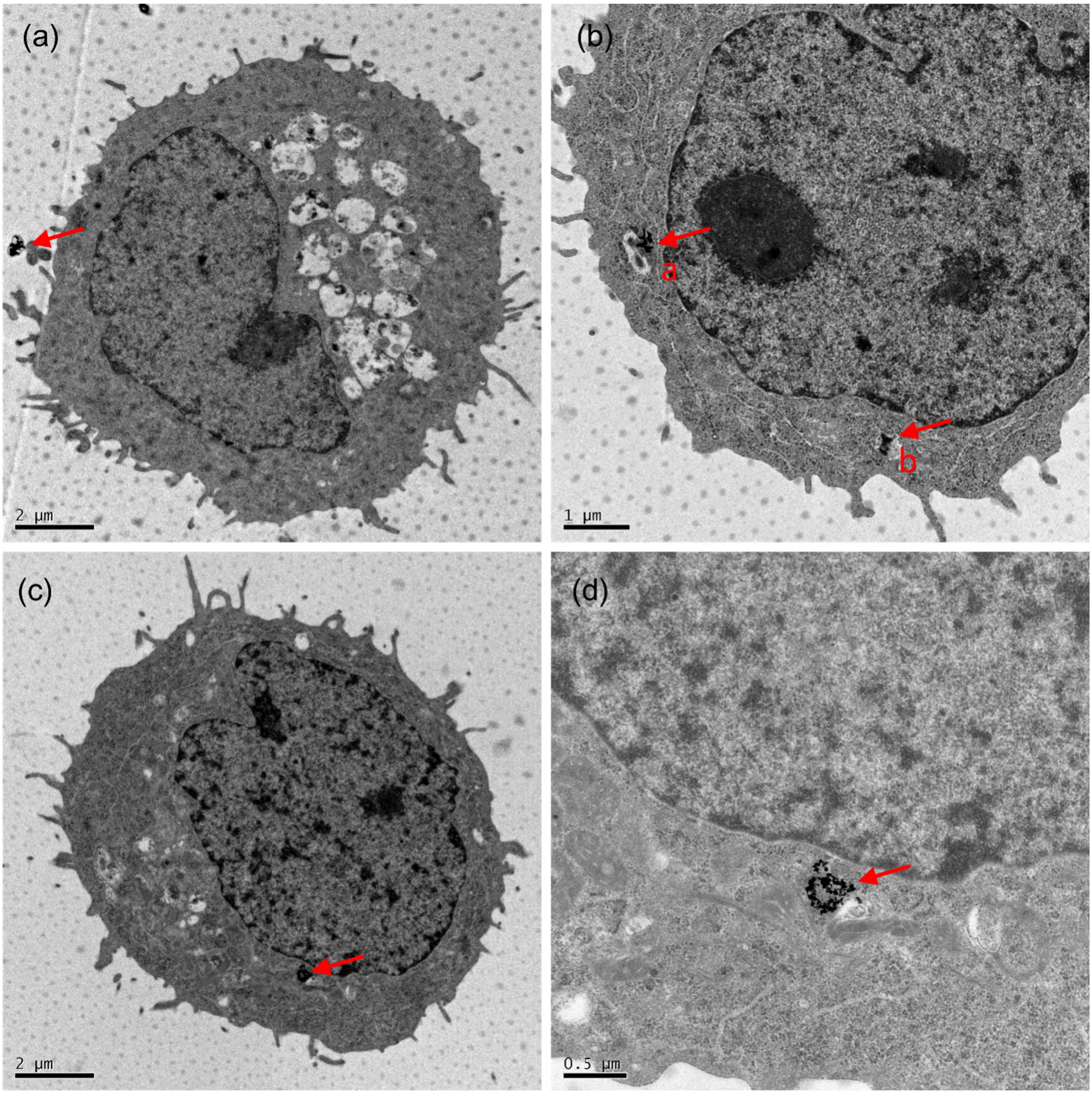

Figure S5. Bio-Transmission electron micrographs show the Hep G2 cells with GNRs. (a) The Bio-TEM image of one Hep G2 cell incubated with GNRs at initial 3th hour. (b), (c), (d) The Bio-TEM image of Hep G2 cell incubated with GNRs after 12 hours with different magnification scale.

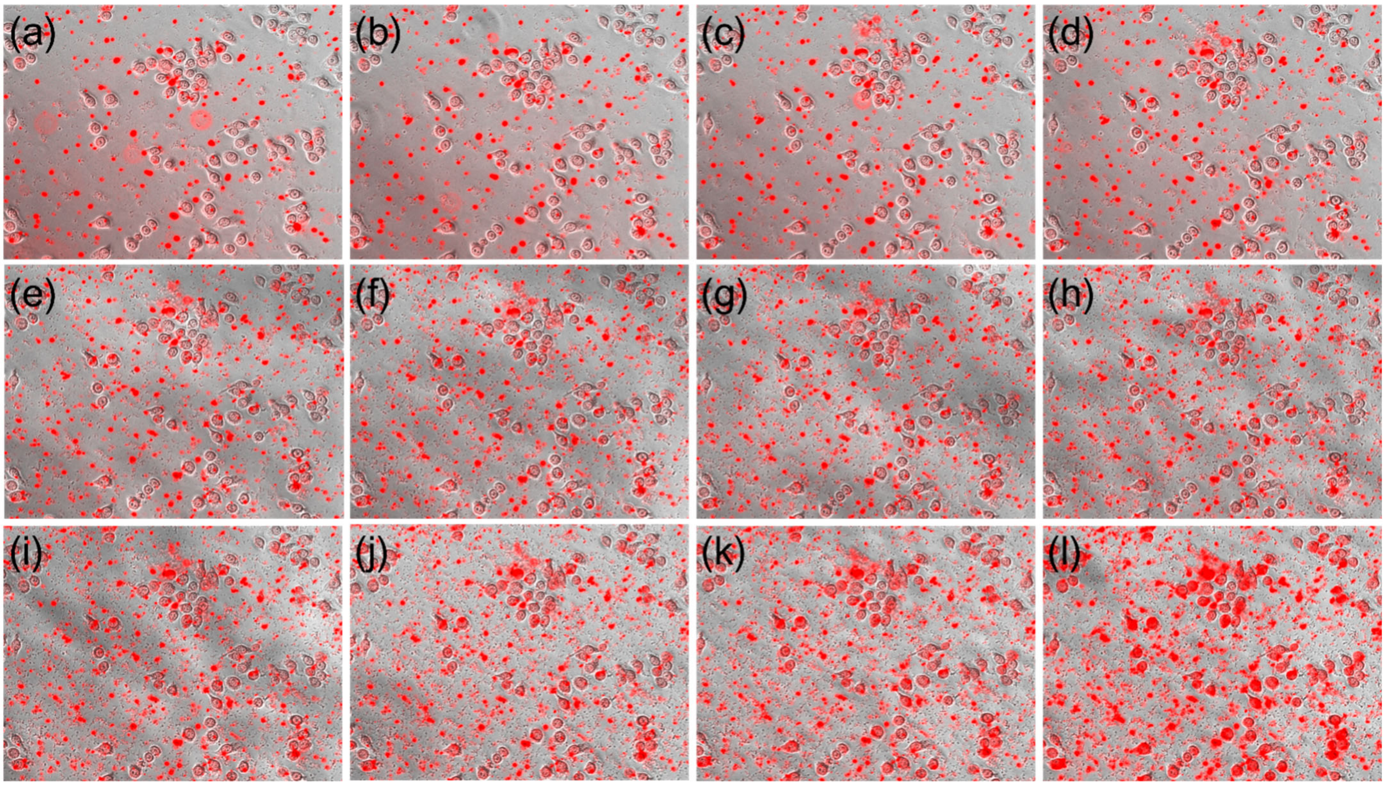

Figure S6. The High content screen (HCS) images of Hep G2 cell incubated with GNRs modified with Cy-5 fluorescent probe. Images from (a) to (l) represents the incubation time of 0 h, 1 h, 2 h, 3 h, 4 h, 5 h, 6 h, 7 h, 8 h, 9 h, 10 h, 11 h, 12 h.

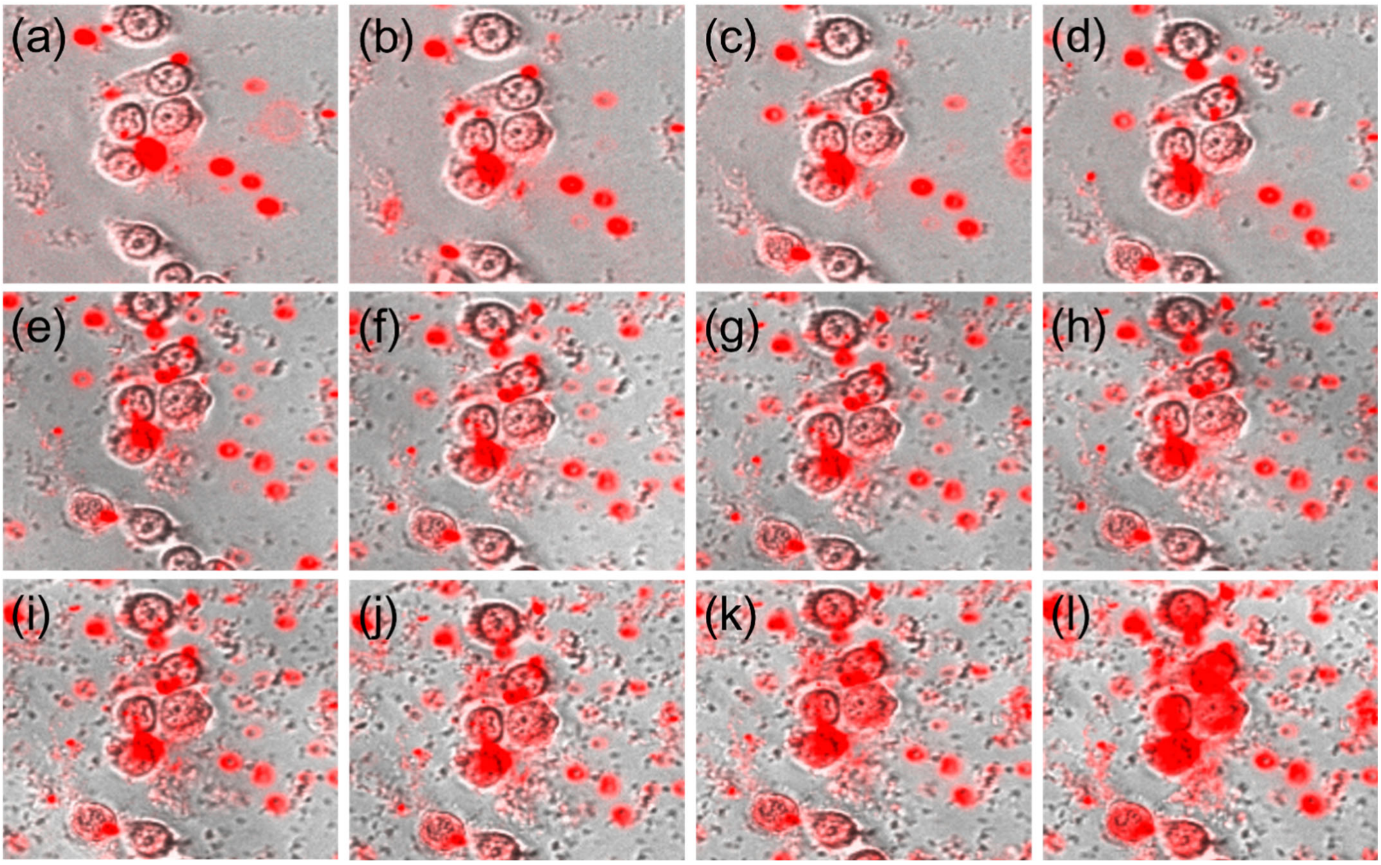

Figure S7. The High content screen (HCS) images of Hep G2 cells incubated with GNRs modified with Cy-5 fluorescent probe. Images from (a) to (l) represents the incubation time of 0 h, 1 h, 2 h, 3 h, 4 h, 5 h, 6 h, 7 h, 8 h, 9 h, 10 h, 11 h, 12 h under larger magnification scale.

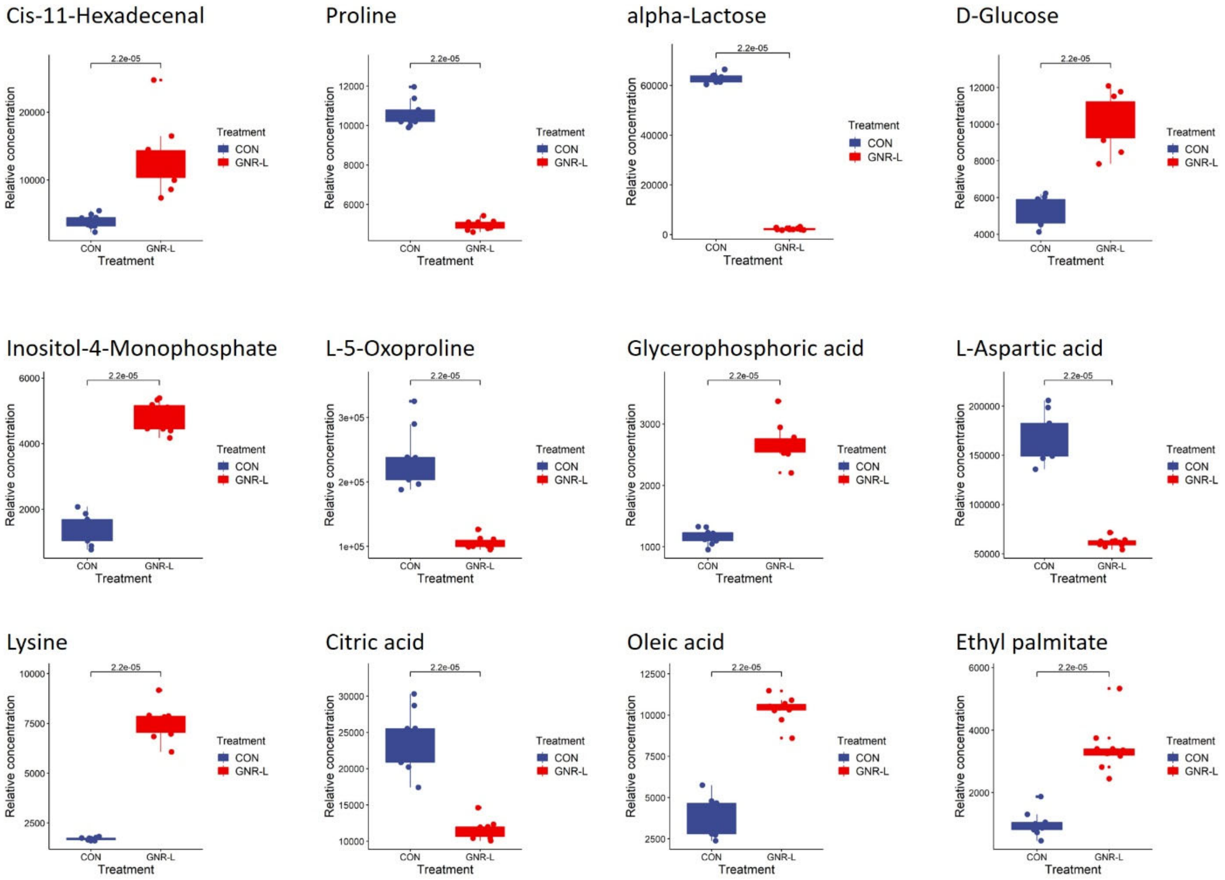

Figure S8. Boxplot of significant changed metabolites in GNR-L dosed group for 12 h
